# The Functional Role of Spinal Interneurons Following Traumatic Spinal Cord Injury

**DOI:** 10.3389/fncel.2020.00127

**Published:** 2020-05-18

**Authors:** Mohammad-Masoud Zavvarian, James Hong, Michael G. Fehlings

**Affiliations:** ^1^Division of Genetics and Development, Krembil Research Institute, University Health Network, Toronto, ON, Canada; ^2^Institute of Medical Science, University of Toronto, Toronto, ON, Canada; ^3^Faculty of Medicine, University of Toronto, Toronto, ON, Canada; ^4^Division of Neurosurgery, University of Toronto, Toronto, ON, Canada

**Keywords:** spinal cord injury, interneurons, synaptopathy, synaptic connections, neuroplasticity

## Abstract

Traumatic spinal cord injury (SCI) impedes signal transmission by disrupting both the local neurons and their surrounding synaptic connections. Although the majority of SCI patients retain spared neural tissue at the injury site, they predominantly suffer from complete autonomic and sensorimotor dysfunction. While there have been significant advances in the characterization of the spared neural tissue following SCI, the functional role of injury-induced interneuronal plasticity remains elusive. In healthy individuals, spinal interneurons are responsible for relaying signals to coordinate both sympathetic and parasympathetic functions. However, the spontaneous synaptic loss following injury alters these intricate interneuronal networks in the spinal cord. Here, we propose the synaptopathy hypothesis of SCI based on recent findings regarding the maladaptive role of synaptic changes amongst the interneurons. These maladaptive consequences include circuit inactivation, neuropathic pain, spasticity, and autonomic dysreflexia. Recent preclinical advances have uncovered the therapeutic potential of spinal interneurons in activating the dormant relay circuits to restore sensorimotor function. This review will survey the diverse role of spinal interneurons in SCI pathogenesis as well as treatment strategies to target spinal interneurons.

## Introduction

Spinal cord injury (SCI) is a heterogeneous disorder that has devastating consequences on the patient’s well-being, independence, and quality of life (National Spinal Cord Injury Statistical Center, 2020). Initiated by either traumatic or non-traumatic aetiologies, SCI constitutes the second leading cause of paralysis worldwide (Armour et al., 2016; Ahuja et al., 2017; Badhiwala et al., 2020). Traumatic SCI results from the compression, contusion, and laceration of the spinal cord due to an external mechanical force. This initial shock produces toxic cellular debris and disrupts the local vasculature, leading to maladaptive consequences, including hypoxia, swelling, and inflammation (Fleming et al., 2006). Referred to as secondary injury, these pathologies exacerbate the spinal cord damage and present a barrier to the patient’s recovery (Tator and Fehlings, 1991). In the clinic, the manifestation of traumatic SCI is classified as either complete or incomplete injury (Blesch and Tuszynski, 2009). Complete SCI leads to the loss of both sensorimotor and autonomic function distal to the injury site. In contrast, incomplete SCI cases continue to possess partial connectivity across the spinal cord, with varying degrees of functional deficits in surrounding neural circuits (Raineteau and Schwab, 2001; Sekhon and Fehlings, 2001).

One of the ambiguous hallmarks of secondary SCI pathophysiology is the induced neuroplasticity in the perilesional neural tissue following complete and incomplete injuries (Hutson and Di Giovanni, 2019). Spinal neuroplasticity refers to the ability of spinal neural circuits to make physiological, anatomical, and functional changes in response to a stimulus (Baker-Herman et al., 2004; Cadotte et al., 2012). The neuroplastic nature of spinal synapses is crucial for the development of neural relay circuits during the embryonic and adolescence stage (Ladle et al., 2007). However, irregular synaptic alterations following injury or disease can lead to pain and spasticity (Cadotte et al., 2012; Cadotte and Fehlings, 2013). A crucial element of neuroplasticity in both healthy and injured spinal cords are interneurons, as they relay signals between different types of neurons to coordinate complex neurotransmission. Although the full extent of neuroplasticity in the injured spinal cord remains unknown, interneurons undergo dramatic changes after injury, which further complicates the injury progression (Harkema, 2008).

The investigation of spinal neural circuits and the underlying interneurons dates back to 1906, when the first paradigm of spinal reflexes was elucidated by Charles Sherrington (Burke, 2006). Further studies by Graham Brown, John C. Eccles, Anders Lundberg, and Elzbieta Jankowska in cat models defined the fundamental basis of spinal circuits and connectivity (Brown, 1914; Eccles, 1968; Jankowska, 2001; Goulding, 2009). These pioneering studies were crucial for describing spinal reflexes and locomotion. Additionally, the concept of a central pattern generator (CPG), which is a connected network of interneurons that function as local executive units to generate neural oscillations and subsequent rhythmic motor activity, was described in these studies (Guertin, 2013).

Current investigations of spinal neural circuits utilize leading-edge technologies to examine neuronal cytoarchitecture, electrophysiological properties, synaptic connectivity, single-cell transcriptional profile, and cell lineage. Emerging technologies, such as RNA sequencing, viral tracing, *in vivo* microscopy, and transgenic manipulations, have revolutionized our understanding of spinal neural circuits. Advances in single-cell RNA-sequencing can determine the cellular identity, molecular markers, and expressional zonation in the spinal cord (Sathyamurthy et al., 2018; Zeisel et al., 2018; Delile et al., 2019). Importantly, targeted genetic manipulations based on these transcriptional profiles elucidate the role of interneuronal subpopulations (Moran-Rivard et al., 2001; Wilson et al., 2008; Talpalar et al., 2013). In parallel, viral tracing provides crucial information regarding connectivity and pathways of individual neurons. Lastly, advanced imaging techniques allow the characterization of cell morphology, location, and lineage in live animals (Sekiguchi et al., 2016). These technologies have characterized the function of different cells involved in neural circuits both in healthy and injured spinal cords. However, the neuron-to-neuron as well as neural-glial connectivity and plasticity during both normal state and injury remains obscure. The vital role of interneurons in both healthy and injured spinal cords is of high interest. This article aims to provide a concise overview of the spinal interneurons heterogeneity and their role following traumatic injury.

## Spinal Interneurons

Healthy spinal neural circuits are comprised of four main classes of local neurons including motoneurons, preganglionic neurons, ascending projection neurons, and spinal interneurons (Zholudeva et al., 2018). Recent single-cell transcriptional analysis has characterized 43 distinct neuronal populations in the rodent lumbar spinal cord (Sathyamurthy et al., 2018). These neurons are surrounded by various glial and vascular cells including mural cells, smooth muscle cells, oligodendrocytes, fibroblasts, endothelial cells, and astrocytes (Vanlandewijck et al., 2018). In addition, the spinal cord houses a variety of multipotent stem cells in the ependymal layer, including oligodendrocyte progenitor cells (OPC) and neural progenitor cells (NPC) (Meletis et al., 2008; Barnabé-Heider et al., 2010).

**Figure 1:**
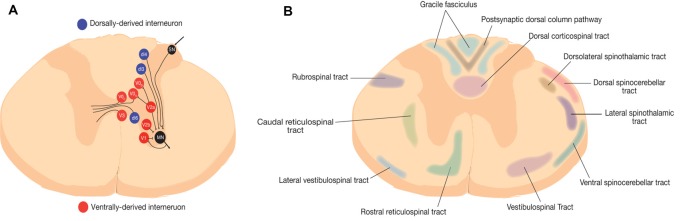
Representative schematic of spinal neural circuits in rodents. **(A)** Spinal interneurons are represented at the thoracic level. Red represents ventrally-derived interneurons and blue represent dorsally derived interneurons (Zholudeva et al., 2018). **(B)** Ascending and descending white matter tracts in rodents (Saliani et al., 2017).

In healthy individuals, spinal interneurons relay sensorimotor input, transduce sensorimotor information sent from the spinal cord to supraspinal centers by ascending tract neurons, modulate motoneuron activity, transmit information between near and distant spinal cord segments, and provide a transmission line to the opposite side of the spinal cord (Figure 1A; Zholudeva et al., 2018). Although the full functional role of spinal interneurons continues to be investigated, they are known to play crucial roles in vital functions, such as breathing through the phrenic circuit or locomotion through CPGs (Guertin, 2013).

Although various classification systems exist to describe the diversity of interneurons present in the spinal cord, they can be broadly classified into local and propriospinal interneurons. Local interneurons encompass short projections, which are crucial for neural oscillations and reflexes. In contrast, propriospinal interneurons are defined as neurons that project to a different spinal segment from which their cell bodies are located (Flynn et al., 2017). The propriospinal interneurons are further subdivided into short propriospinal interneurons and long propriospinal interneurons. Short propriospinal interneurons provide intersegmental connections, whereas long propriospinal interneurons connect distant regions, for instance between cervical and lumbar spinal regions. These propriospinal interneurons are crucial for interlimb coordination and play an important role in generating new circuits following injury (Flynn et al., 2011; Laliberte et al., 2019).

Local interneurons project either ipsilaterally or contralaterally. The interneurons that project to the opposite side of the spinal cord are referred to as contralateral, whereas interneurons that project into the same side are referred to as ipsilateral. The interneurons that project through the midline are referred to as commissural interneurons. Inhibitory interneurons release inhibitory neurotransmitters, such as γ-aminobutyric acid (GABA) and glycine. Whereas, excitatory interneurons release excitatory neurotransmitters, such as glutamate (Goulding, 2009).

Spinal interneurons are also classified based on their location and transcriptional profile during development, which arise from seven dorsal and four ventral progenitor cells (Table 1). Further characterization of the subtypes for each of these neuronal populations enables a reliable identification scheme to define their projection patterns, released neurotransmitters, and connectivity. During gestation, neuroepithelial cells at the ventricular zone of the neural tube are under the influence of a two-dimensional morphogen gradient system between the floor plate and the roof plate, which lead to the formation of several progenitor cells with differential expression of homeodomain transcription factors. These crucial morphogens include sonic hedgehog (Shh) and bone morphogenic proteins (BMP). In addition, the opposing fibroblast growth factor (FGF) and retinoic acid (RA) signalling molecules guide the rostral and caudal characterization of cells respectively (Jessell, 2000; Lu et al., 2015; Ogura et al., 2018). The result is the morphogenesis of 23 types of neurons through a series of cell divisions, which includes eight dorsally-derived interneurons, 13 ventrally-derived interneurons, and the connected motor neurons (Figure 1).

**Table 1:** The list of presently identified spinal interneurons and their progenitor pool.

Progenitor zone	Cell type	Neurotransmitter
Pd1	Dl1ic	Glutamate
	Dl1i
P2	Dl2	Glutamate
Pd3	Dl3	Glutamate
Pd4	DI4	GABA
PdIL	dILA	GABA
	dILB	Glutamate
Pd5	Dl5	Glutamate
Pd6	Dl6	GABA/Glycine
P0	V0_D_	GABA/Glycine
	V0_V_	GABA/Glycine
	V0_C_	Acetylcholine
	V0_G_	Glutamate
P1	Renshaw	Glycine/GABA
	Ia	Glycine
	V1	Glycine/GABA
P2	V2a	Glutamate
	V2b	GABA/Glycine
	V2c	GABA/Glycine
	V2d	Glutamate
pMN	Mn	Acetylcholine
P3	Vx	Glutamate
	V3d	Glutamate
	V3v	Glutamate

### Dorsally-Derived Interneurons

There are eight classes of dorsally-derived interneurons, dI1 to dI6 as well as dILA and dILB, arising from seven progenitor cells (Lu et al., 2015). Although these interneurons originate from the dorsal region, some migrate ventrally during development. These cells are generated sequentially in early, mid, and late phases. The early phased interneurons include dI1 to dI3 and are generated from pd1 to pd3 progenitor cells, respectively. These cells migrate ventrally after development. The mid phase dorsal dI4 to dI6 are generated from pd4 to pd6 cells. Lastly, the late phase dILA and dILB are generated from pdIL progenitors cells (Gross et al., 2002; Lu et al., 2015).

The early-phased pd1, pd2, and pd3 progenitor cells express Atoh1, Ngn1/2, and Nash1 basic-helix-loop-helix (bHLH) transcription factors, respectively (Gowan et al., 2001; Avraham et al., 2009; Duval et al., 2014). The differentiated interneurons from these progenitors are characterized based on their LIM-homeodomain (LIM-HD) transcription factors, which mediate their axonal guidance and projection (Avraham et al., 2009). Specifically, the dI1 interneurons express Lhx2/9, while dI2 and dI3 interneurons express Lhx1/5 and Isl1, respectively (Gross et al., 2002; Avraham et al., 2009). The *dI1* interneurons are located in the dorsal horn and integrate proprioceptive signals from the peripheral organs and project rostrally to the spinocerebellar tract (Gross et al., 2002; Avraham et al., 2009). These neurons can be further subdivided into contralaterally-projecting dI1c and ipsilaterally-projecting dI1i interneurons (Wilson et al., 2008). *Lhx2/*9 mutations result in loss of mid-line crossing in dI1c interneurons, which demonstrates their role in defining subtype identity in these neurons (Wilson et al., 2008). The dI2 interneurons are located in the intermediate spinal cord and the ventral horn, which are speculated to project contralaterally to transmit sensory input to the thalamus *via* the spinothalamic tract (Figures 1A,B; Gross et al., 2002). The dI3 interneurons are located in the intermediate spinal cord and the dorsal horn (Bui et al., 2013). These are excitatory neurons that project rostrally, ipsilaterally, and longitudinally through monosynaptic connections (Stepien et al., 2010; Bui et al., 2013; Lu et al., 2015).

The mid-phased dI4 to dl6 interneurons express Lbx1. In *Lbx1* knock out animals, there is disrupted sensory transmission and excessive generation of commissural neurons (Gross et al., 2002). The *dI4* interneurons are inhibitory GABAergic neurons, which are located in the dorsal horn and project ipsilaterally to convey somatosensory information (Gross et al., 2002). The dI5 interneurons are located at the dorsal horn, which are glutamatergic and contralaterally projecting neurons (Gross et al., 2002). These neurons express Brn3a, Tlx1, Tlx3, and Lmx1b transcription factors (Gross et al., 2002). The dI6 interneurons are located in the ventromedial spinal cord and are subdivided to neurons with either Wt1 or Dmrt3 expression (Gosgnach et al., 2017). Wt1 eletion alters forelimb hindlimb coordination in mice (Schnerwitzki et al., 2018). In contrast, Dmrt3 knock out mice exhibit altered stride length and swing time (Andersson et al., 2012).

The dILA and dILB develop later from a common progenitor cell, and their cell fate is significantly influenced by Achaete-Scute Family BHLH Transcription Factor 1 (ASCL1; Mizuguchi et al., 2006). These interneurons are mainly located in the superficial dorsal horn and carry out different roles in spinal circuits. The dILA interneurons are GABAergic, whereas dILb are glutamatergic and demonstrate a differential transcriptional profile from each other (Mizuguchi et al., 2006; Lu et al., 2015).

### Ventrally-Derived Interneurons

The ventrally-derived interneurons arise from *four* progenitor cells called p0, p1, p2, and p3. Further maturation of these three lines of interneurons gives rise to a series of interneurons crucial for locomotion (Kiehn, 2016). V0 interneurons are characterized by their expression of Dbx1 (Pierani et al., 2001; Lanuza et al., 2004). These neurons are comprised of four subpopulations, including ventral (*V0_V_*), dorsal (*V0_D_*), cholinergic (*V0_C_*), and glutamatergic (*V0_G_*; Zagoraiou et al., 2009). The ablation of V0 interneurons or the deletion of Dbx1 leads to altered left-right coordination (Lanuza et al., 2004; Talpalar et al., 2013). The V0_V_ and V0_D_ are distinguished based on the expression of Evx1 and Pax7 factors (Talpalar et al., 2013). V0_V_ neurons are excitatory and project commissural and play an important role in controlling locomotion (Talpalar et al., 2013).

The V1 interneurons are a large heterogenous class of inhibitory neurons, which include the vital renshaw and Ia interneurons (Bikoff et al., 2016). These neurons project ipsilaterally, express Engrailed1 (En1), and are crucial for executing of rhythmic activity *via* their function in recurrent and reciprocal inhibition (Falgairolle and O’Donovan, 2019). Recurrent inhibition (also known as Renshaw inhibition) refers to the suppressive effect of Renshaw cells back on the motoneurons that initiated their activation in a negative feedback mechanism (Özyurt et al., 2019). In contrast, reciprocal inhibition refers to the action of Ia interneurons to inhibit of antagonist neurons during the activation of the agonist neuron (Crone et al., 1987; Katz et al., 1991). Genetic inhibition of En1 expressing interneurons in mice demonstrates the importance of these neurons to generate rhythmic activity and quick locomotion (Gosgnach et al., 2006). A subsequent genetic ablation study in zebrafish echoed these results by demonstrating that V1 interneurons are crucial for suppressing the activities of V2a and motor neurons (Kimura and Higashijima, 2019).

The V2 interneurons are derived from P2 progenitor cells and express LIM Homeobox 3 (Lhx3) transcription factor. These neurons project ipsilaterally and consist of four subpopulations, including V2a, V2b, V2c, and V2d (Dougherty et al., 2013; Harris et al., 2019). V2a are excitatory neurons and are characterized by the expression of Chx10 and are important in locomotion and left-right coordination. The V2a ablation disrupts left-right coordination (Crone et al., 2008, 2009; Dougherty and Kiehn, 2010). These interneurons are further classified into type 1 and type 2 groups, with varying projections and transmission patterns (Hayashi et al., 2018; Menelaou and McLean, 2019). Single cell RNA-seq experiments reveal that these subtypes each encompass multiple subpopulations (Hayashi et al., 2018). The V2b interneurons are inhibitory and express Gata2/3 (Zhang et al., 2014). The V2c function is still unknown, but these cells have been shown to express Sox1 and do not express Gata3 (Lu et al., 2015; Harris et al., 2019). The V2d cells are newly characterized excitatory interneurons (Dougherty et al., 2013; Harris et al., 2019).

The V3 interneurons are glutamatergic, predominantly commissural, and necessary for locomotion (Zhang et al., 2008). They originate from the ventral Nkx2.2 + p3 progenitor domain, which is defined by a marked expression of the transcription factor Sim1. A recent study (Deska-Gauthier et al., 2020) elucidated that V3 interneurons are organized into early and late-born neurogenic waves, where late-born neurons displayed restricted sub-population fates (Deska-Gauthier et al., 2020). Further, the Sim1 transcription factor, while expressed on all post-mitotic V3 interneurons, is critical only for the diversification of early-born, but not late-born, V3 interneurons. This suggests that the timing of neurogenesis can alter the way definitive transcription factors regulate subpopulation cell fate specification. To date, several studies have characterized the function of V3 interneurons and concluded that they play a significant role in spinal locomotor circuitry and gait transitions through direct connections with the rhythm-generating circuits (Rybak et al., 2006; Danner et al., 2019). Strikingly, these studies have shown that V3 interneurons do not form synapses with motoneurons; however, contradicting data from anatomical studies have illustrated boutons from these neurons in apposition to motoneurons (Zhang et al., 2008). This, in conjunction with developmental studies that demonstrate the existence of several dorsal and ventral sub-populations of V3 interneurons, suggests these conflicting observations may be due to the functional diversity of V3 interneuron sub-populations. Indeed, a recent study using patch-clamp and glutamate uncaging demonstrated that V3 interneurons form functional layers (Chopek et al., 2018). The ventromedial V3 interneurons form synapses with each other, whereas the ventrolateral V3 interneurons form synapses with the ipsilateral motoneurons. These motoneurons then provide excitatory and glutamatergic feedback to the V3 interneurons. This distinct circuitry implicates that V3 interneurons can contribute to precise spatiotemporal movements.

## Injury-Induced Neuroplasticity

**Figure 2:**
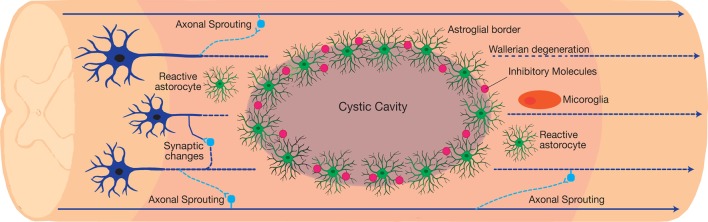
The neuroplastic nature of the spinal cord following traumatic incomplete spinal cord injury (SCI). The injury results in the formation of three distinct histological compartments: a non-neuronal lesion core, an astroglial scar, and a spared reactive perilesional neural tissue. Each compartment consists of a unique cellular composition and exhibits a distinct pathophysiology after injury. In the perilesional zone, reactive glia and the induced neuroplasticity alters the neuronal connections. Axonal sprouting and plasticity form bypass routes around the lesion, resulting in limited functional recovery after injury. However, maladaptive synaptic changes contribute to the dormancy of the spared neural circuits around the lesion, which introduces a barrier to recovery.

Although traumatic SCI culminates in demyelination, cavitation, glial reactivity, and impaired neural connections, many patients demonstrate functional improvements following incomplete injury (Fawcett et al., 2007). Recent investigations on SCI pathogenesis have elucidated the mechanisms behind functional improvements and barriers to recovery (Courtine and Sofroniew, 2019). There are three distinct histological compartments formed after injury, including a non-neuronal lesion core, astroglial border, and reactive prelisional neural tissue (Figure 2; O’Shea et al., 2017). Each compartment consists of distinctive cellular composition and undergoes unique pathophysiological changes after injury (Sofroniew, 2018).

In the injury epicenter, injury-induced cell death introduces toxic debris into the spinal cord, which recruits both the local microglia and the circulatory immune cells to clear the introduced debris. Although this immune reaction initially plays a beneficial role, the extended reactivity of immune cells will further damage the neural tissue. The astroglial border is crucial for controlling the spread of immune cells. Serum proteins and local cell markers including ATP, Shh, BMPs, thrombin, FGF, and endothelin, promote astrocyte proliferation. This is in parallel to the synthesis and secretion of chondroitin sulfate proteoglycans (CSPGs) *via* NG2 OPC (NG2-OPC) and macrophages into the extracellular matrix (Jones et al., 2002). The newly formed astrocytes migrate and intermingle with CSPGs in order to organize into a barrier immediately adjacent to the non-neural lesion core, dubbed the astroglial scar (Silver and Silver, 2014). While the functional role of this barrier and the reactive glial cells after injury continues to be under investigation, the barrier is known to actively inhibit axonal regeneration and repair through the covalently attached chondroitin sulfate glycosaminoglycan (CS-GAG) side-chains of CSPGs (Anderson et al., 2016; Burnside et al., 2018; Bradbury and Burnside, 2019).

The glial reaction to trauma extends rostro-caudal from the astroglial scar into the perilesional zone of spared neural tissue. Within this region, astrocytes, microglia, and NG2-OPC induce spontaneous synapse and circuit reorganization. In contrast to the newly formed astrocytes in the astroglial scar border, hypertrophic reactive astrocytes in the perilesional zone maintain their connections with local neurons (Khakh and Sofroniew, 2015). The activity of reactive glial cells gradually reduces and transitions into healthy spinal cord tissue. Synapse loss in the perilesional zone spontaneously leads to formation of new synapses. Damaged axons either retract or sprout and form new synapses around the lesion core through the spared neural tissue (Figure 2). Axonal sprouting in propriospinal interneurons has the ability to form a detour around the lesion core, and is a likely mechanism for endogenous behavioural improvement following incomplete traumatic SCI (Bareyre et al., 2004; Ballermann and Fouad, 2006; Courtine et al., 2008; Takeoka et al., 2014). This is particularly evident in the C2 hemisection SCI model aimed at investigating respiratory dysfunction after injury. Within this model, respiration is restored *via* the activity of propriospinal interneurons that form new connections, referred to as crossed phrenic phenomenon (CPP).

## Synaptopathy Hypothesis

The International Campaign for Cures of Spinal Cord Injury Paralysis (ICCP)—tasked at reviewing SCI clinical trials in 2007—concluded that most SCI patients suffer from a complete functional loss distal to the injury level (Grade A by the American Spinal Injury Association; ASIA). These patients show minimal recovery after 1 year, despite having a rim of preserved neural tissue around the injury site. This suggests that either remyelination or synaptic readjustment can elicit improved recovery. Surprisingly, all other ASIA classifications (Grades B, C, and D) demonstrate behavioral improvements, but the majority of Grade A patients do not exhibit any improvements 1-year post-injury (Adams and Cavanagh, 2004; Fawcett et al., 2007; Steeves et al., 2007; Tuszynski et al., 2007). In the post-ICCP era, the Spinal Trials Understanding, Design, and Implementation (STUDI) group—summoned in 2018—identified the role of combinatorial and rehabilitative trainings in ongoing clinical trials (Curt, 2019). This highlights the potential for neuroplasticity following injury and illustrates the growing interest in utilizing the neuroplastic potential of the spinal cord to treat SCI patients.

The synaptopathy hypothesis of SCI combines the recent findings from our lab and others, which suggests that traumatic SCI disrupts the preserved synaptic connections amongst the spinal interneurons. Despite the adaptive role of axonal sprouting in endogenous recovery, overwhelming evidence suggests neurotransmitter imbalance following SCI in the perilesional area results in an imbalanced excitatory/inhibitory (E/I) ratio, leading to the functional inactivation of preserved tissue (Chen et al., 2018). Synaptopathy is defined as maladaptive synaptic alterations, which lead to dysfunctional neurocircuitry. Various neurological conditions, such as autism spectrum disorder (ASD), schizophrenia, epilepsy, and Alzheimer’s disease have been linked to synaptic alterations (Ko et al., 2015).

Electrophysiological analyses using *in vivo* whole-cell patch clamp in a rat hemisection model demonstrate elevated spontaneous action potential firing in caudal substantia gelatinosa neurons following injury (Kozuka et al., 2016). While the mechanism remains to be fully elucidated, this hyperexcitation suggests the elimination of tonic descending control of inhibitory spinal interneurons (Kozuka et al., 2016). Interestingly, rehabilitative and neuromodulatory treatments aimed at exploiting synaptic changes after injury can activate dormant neural pathways (Petruska et al., 2007; Darrow et al., 2019; Kobayakawa et al., 2019). Transcriptional analysis in injured rats demonstrates the differential expression of synaptic genes following rehabilitative training (Kobayakawa et al., 2019). A recent study utilizing combinatorial rehabilitative training and Epidural stimulation (EDS) demonstrated that complete SCI patients, classified as grade A under ASIA score, were able to regain walking ability following several treatment sessions. Surprisingly, the patients lose their functional improvements following the cessation of the EDS. This is indicative of the beneficial role of neuromodulatory treatments to balance neurotransmission and activate dormant circuits after injury (Angeli et al., 2018).

Irregular neurotransmitter production, clearance, and sensitivity are potential mechanisms for maladaptive synaptic changes following traumatic SCI (Liu et al., 2004; Huang et al., 2016). For instance, altered serotonin regulation following injury is attributed to functional deficits, such as spasms (Thaweerattanasinp et al., 2020). Additionally, increased clustering of 5-HT2C receptors on V2a interneurons results in the super-sensitivity of neurons to serotonin following SCI (Husch et al., 2012).

A subtype of synaptopathy is channelopathy—the improper function of ion channels—which are critical for synaptic transmission and signal propagation in neural tissue (Hanna, 2006). Traumatic SCI alters the expression of ligand-gated channels, which in turn fluctuates the physiology of synapses in spinal interneurons. For instance, K^+^/Cl^−^ cotransporter type 2 (KCC2) is a neuron specific transmembrane protein responsible for maintaining chloride and potassium ion gradients in the synaptic cleft (Moore et al., 2019). Previous studies have demonstrated the involvement of KCC2 in SCI pathogenesis. Expressed by SLC12A5, reduced KCC2 expression after injury lowers the concentration of chloride ions in the extracellular matrix (Boulenguez et al., 2010). The opening of chloride channels by GABA will induce chloride outflux from cells, which induces an excitatory effect. Such synaptic change upstream of inhibitory interneurons results in increased inhibition after SCI, which impairs signal transduction across spared neural tissue (Chen et al., 2018).

## Spinal Interneurons as a Therapeutic Target After SCI

**Figure 3:**
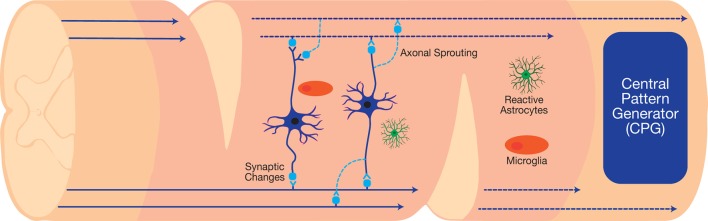
The staggered double hemisections (SDH) model interrupts all supraspinal connections while sparing spinal interneurons, which can be used to investigate neuroplasticity after SCI.

Therapeutic interneuronal modulation seeks to either reverse adverse maladaptive synaptic changes following injury or form new circuits to bypass the damaged injury site. The clinical translation of such therapeutics can restore vital autonomic functions, such as respiration, after high cervical injuries (Golder and Mitchell, 2005; Satkunendrarajah et al., 2018). In preclinical studies, the staggered double hemisections (SDH) SCI model enables the examination of interneuronal connections in the absence of supraspinal inputs (Figure 3). This model suspends all supraspinal inputs by two contralateral hemisection incisions, but spares relay circuits in between the incision sites (Laliberte et al., 2019). Various types of studies on the SDH model have developed therapeutics to target relay circuits and interneurons. Thus far, these include EDS, rehabilitative training, as well as molecular interventions *via* pharmacological and gene therapy approaches.

### Epidural Stimulation and Physical Rehabilitation

EDS refers to the introduction of electrical current *via* an electrode implanted over dura. Combined electrochemical neuroprosthesis and rehabilitation through treadmill-aid/robotic postural interface enabled the cortical control of locomotion through spared neural tissue (Lavrov et al., 2008; Angeli et al., 2018; Capogrosso et al., 2018). While effective in reactivating locomotor circuitry, this stimulation is not exclusive to spinal interneurons—but recent work has shown that the activation of sensory afferents recruits interneurons and motoneurons to produce coordinated hindlimb stepping (Formento et al., 2018). This, in conjunction with the functional recovery gained after SDH from the use of a pharmacological agent—quipazine—that directly targets propriospinal interneurons, is compelling evidence that propriospinal relay is critical for EDS-mediated functional recovery (Gerasimenko et al., 2007).

### Pharmacological and Gene Therapy Approaches

The currently evolving gene therapeutic techniques, as well as pharmacological interventions, demonstrate new ways to influence neuroplastic change in spinal interneurons (Dimidschstein et al., 2016; Wang et al., 2019). These techniques are particularly crucial for restoring breathing following damage to the phrenic circuit. For instance, the pharmacogenetic stimulation of mid-cervical excitatory interneurons demonstrates improved breathing in mice after C2 hemisection injury with disrupted ipsilateral bulbospinal connections (Satkunendrarajah et al., 2018). In parallel, an alternative approach is to restore E/I balance amongst the interneurons to mitigate maladaptive synaptic alterations after injury. Particularly, virally-induced episomal expression of KCC2 in GABAergic neurons significantly improves the activity of propriospinal interneurons between the SDH sites and augments hindlimb functional recovery (Chen et al., 2018). Similarly, the administration of either the glycinergic antagonist strychnine or the GABA_A_ receptor antagonist bicuculline improves stepping in the absence of rehabilitative training (Robinson and Goldberger, 1986; de Leon et al., 1999). While these aforementioned studies targeted the cervical and thoracic regions of the spinal cord, studies that investigated the role of interneurons below in the lumbar region using optogenetics demonstrated that V2A (Ljunggren et al., 2014) and Shox2+ (Dougherty et al., 2013) interneurons are critical for stepping and rhythmogenesis within the lumbar locomotor CPGs. Taken together, these studies demonstrate that both activation of excitatory and modulation of inhibitory interneurons may be critical for functional recovery.

## Conclusions

The current clinical practice for traumatic SCI is limited to prevention and early neuroprotection (Chio et al., 2019). Several emerging treatment strategies, such as anti-NOGO treatment (currently at phase 2/3 clinical trial) are being developed (Kucher et al., 2018). These treatments are able to improve behavioral outcome measures, but not fully recover lost function. The heterogeneity of SCI warrants the development of combinatorial approaches to treat SCI patients. An important consideration in such studies is the role of interneurons and synaptic modifications to activate preserved host tissue. Further investigations into the mechanisms behind synaptic changes in the injured spinal cord will improve the available treatment strategies for SCI patients.

## Author Contributions

M-MZ and JH: literature review, manuscript writing, editing, and finalizing, approval of the final manuscript. MF: framing the concept and structure of the review, editing, and finalizing, approval of the final manuscript.

## Conflict of Interest

The authors declare that the research was conducted in the absence of any commercial or financial relationships that could be construed as a potential conflict of interest.
